# Hard Gelatin Capsules Compounded and Dispersed in Water in Pediatrics: Real Versus Theoretical Dose Administered

**DOI:** 10.3390/ph19040534

**Published:** 2026-03-25

**Authors:** Romain Paoli-Lombardo, Nicolas Primas, Clémence Tabélé, Ikram Zaddam, Eya Iben Slimene, Pascal Rathelot, Patrice Vanelle, Caroline Castera-Ducros, Christophe Curti

**Affiliations:** 1 Service Central de la Qualité et de l’Information Pharmaceutiques (SCQIP), Pharmacy Department, Assistance Publique-Hôpitaux de Marseille, 174 Boulevard Baille, 13005 Marseille, France; romain-alexandre.paoli-lombardo@ap-hm.fr (R.P.-L.); zaddamikram1@gmail.com (I.Z.); eya.iben-slimene@etu.univ-amu.fr (E.I.S.); pascal.rathelot@ap-hm.fr (P.R.); patrice.vanelle@ap-hm.fr (P.V.); caroline.castera@ap-hm.fr (C.C.-D.); 2 Equipe Pharmaco-Chimie Radicalaire, UMR 7273, Institut de Chimie Radicalaire ICR, CNRS, Aix Marseille Université, 27 Boulevard Jean Moulin, 13385 Marseille, France; 3 Health Services and Quality of Life Research, School of Medicine, Aix-Marseille University, 27 Boulevard Jean Moulin, 13385 Marseille, France; clemence.tabele@ap-hm.fr

**Keywords:** pediatric practice, hard gelatin capsules, drug compounding, dose administered, HPLC-UV

## Abstract

**Background**: In pediatric practice, dose individualization often requires the manipulation of solid oral dosage forms, such as dispersing capsules in water and administering only part of the volume. Despite its frequent use, this practice is poorly documented and may lead to inaccurate dosing. Objectives: This study aimed to assess the actual dose administered when compounded hard gelatin capsules are dispersed in water and partially withdrawn, and to evaluate the influence of different manipulation protocols on dose recovery. **Methods**: Ten active pharmaceutical ingredients (APIs) routinely compounded as pediatric hard gelatin capsules were studied. Content uniformity was first verified according to European Pharmacopoeia (EP) requirements. One capsule was dispersed in 2 mL of water, and 1 mL was withdrawn using three protocols: (1) no mixing, (2) gentle manual mixing with immediate sampling, and (3) gentle manual mixing followed by a 10 s resting period before sampling. Drug content in the withdrawn volume was quantified using validated HPLC-UV methods. Results are expressed as the mean percentage of the theoretical dose ± standard deviation. **Results**: All capsules complied with EP content uniformity criteria. However, partial volume administration resulted in marked and protocol-dependent deviations from the theoretical dose. Without mixing, recovered doses ranged from 17% to 58% of the target dose, with high variability. Gentle mixing improved dose recovery, particularly for APIs forming solutions, such as captopril, thiamine hydrochloride, and clonidine hydrochloride, which achieved values close to 90%. In contrast, APIs forming suspensions consistently resulted in underdosing, even after mixing, with further reductions observed after a short resting period, indicating rapid sedimentation. **Conclusions**: Fractional administration of dispersed hard gelatin capsules leads to unpredictable and often clinically relevant underdosing, especially for poorly soluble APIs. Whenever possible, capsules should be compounded at the prescribed dose, and liquid formulations should be preferred when dose fractionation is required.

## 1. Introduction

In pediatric practice, the content of commercially available drugs often does not correspond to the theoretical required content. Therefore, drugs are manipulated to achieve the required dose [1,2,3,4,5,6]. A recent study at Oslo University Hospital showed that 17% of 3070 oral medication administrations to hospitalized children involved manipulation [1].

For example, dry oral formulations (tablets, capsules) can be dispersed in water, and only part of the total volume can be administered to obtain a lower dose. However, this practice is not supported by any guidelines or evidence [2,3].

Among the few existing studies, one deals with the oral administration of nifedipine in children [4]. The authors first proved that the in-use stability of extemporaneous nifedipine suspension decreases with light exposure. Their results also showed that a patient could receive a dose ranging from 58 to 150% of the theoretical content, depending on the volume of liquid used and the theoretical content, but also on the dexterity of the persons administering the medication.

Two studies on the real versus the theoretical content of aspirin manipulated prior to pediatric administration have also been described [5,6]. In the first study, aspirin tablets were split into halves and quarters, then dispersed in a liquid. The amount recovered ranged from 12% to 99%, depending on the manipulation protocol (medicine measure or oral syringe and rinsing or not), the commercial aspirin drug used (conventional, dispersible, chewable) and the division protocol (half or quarter fragments) [5]. In the second study, the authors investigated the uniformity of doses from samples prepared from dispersible aspirin tablets. They found that the dose administered varied depending on whether the dispersed samples were taken from the bottom or the top of the container, with the dose taken from the top potentially being 23% to 80% lower than the intended dose [6]. However, we decided to focus on another frequent pediatric practice, hard gelatin capsule dispersion in water and partial volume administration to achieve the required individual dosage.

Drug compounding is another common pediatric practice. Hospital pharmacists produce medications tailored to each patient when commercial medicines are not available. Currently, there is a significant trend toward innovation in pediatric compounding practices, such as 3D-printed medication [7,8], mini-tablets or orodispersible tablets [9]. However, these interesting possibilities are not always realistic in real-life hospital practice, both in low- and middle-income countries. In France, for example, in order to ensure safe administration, two compounding solutions coexist, each with advantages and inconveniences: liquid oral formulations and solid oral formulations [10].

Solid oral formulations have the advantage of having a fixed dosage, a better stability and a simple formulation. They can be prepared without any excipients with known action or effect, such as preservatives. Moreover, hard gelatin capsules used in pediatrics can contain only the Active Pharmaceutical Ingredient (API) and one or two fillers such as microcrystalline cellulose, lactose or mannitol.

Hard gelatin capsules are routinely produced by our centralized compounding department. We decided to investigate what happens when these capsules are dispersed in 2 mL of water and only 1 mL is administered to a pediatric patient. Dispersion volume was selected at 2 mL because this volume reflects our current clinical practice and was also suggested in the literature as the highest volume to be orally administered to an infant or toddler for liquid drugs [11].

Ten different APIs were evaluated: clonidine hydrochloride 20 µg, phenobarbital 20 mg, thiamine hydrochloride 50 mg, captopril 3 mg, sildenafil citrate 2 mg, spironolactone 2.5 mg, amiodarone hydrochloride 5 mg, ursodesoxycholic acid 20 mg, nicardipine hydrochloride 0.5 mg and furosemide 2 mg. These APIs and their nominal contents correspond to the hard gelatin capsules routinely compounded in our hospital for administration to pediatric patients.

Table 1 compares the solubility of the drugs studied, as described in the corresponding monographs of the European Pharmacopoeia, and their theoretical content of one capsule in 2 mL of water. Based on these results, we can assume that when hard gelatin capsules containing 20 mg of phenobarbital, 2.5 mg of spironolactone, 5 mg of amiodarone hydrochloride, 20 mg of ursodesoxycholic acid, 0.5 mg of nicardipine hydrochloride, and 2 mg of furosemide are dispersed in 2 mL of water, this will result in suspension and a risk of sedimentation. In contrast, dispersing 20 µg of clonidine hydrochloride, 50 mg of thiamine hydrochloride, 3 mg of captopril, and 2 mg of sildenafil citrate will likely result in a solution and a lower risk of partial dose administration.

However, these theoretical results must be confirmed and corroborated by analytical data.

## 2. Results

All compounded hard gelatin capsules complied with the European Pharmacopoeia requirements for content uniformity. The mean capsule content ranged from 93.55 ± 3.16% to 107.68 ± 5.84% of the nominal dose, with relative standard deviations of less than 6% for all APIs (Table 2). These results confirmed the homogeneity of the compounded batches, the accuracy of our dosing methods and ensured that any deviation observed during partial volume administration was not related to the quality of the capsules themselves.

For the assessment of partial volume administration, the theoretical dose expected in 1 mL after dispersing one capsule in 2 mL of water was defined as the reference value (100%).

For all APIs, the dose recovered in 1 mL differed from the target value of 100%, depending on both the API and the manipulation protocol applied (Table 2).

When 1 mL of the supernatant was collected without mixing (protocol 1), the recovered dose was consistently below the target value for all APIs. The mean recovered doses ranged from 17.18 ± 11.88% for phenobarbital to 57.66 ± 12.08% for thiamine hydrochloride. Significant underdosing was observed for most APIs, particularly for phenobarbital (17.18 ± 11.88%), furosemide (20.87 ± 9.04%), spironolactone (20.94 ± 6.14%), ursodeoxycholic acid (21.16 ± 6.48%), nicardipine hydrochloride (22.77 ± 8.81%), and amiodarone hydrochloride (25.11 ± 8.30%). Slightly higher but still suboptimal recovered doses were observed for clonidine hydrochloride (31.36 ± 11.06%), sildenafil citrate (39.64 ± 11.62%), captopril (41.40 ± 10.72%), and thiamine hydrochloride (57.66 ± 12.08%). High standard deviations were observed for most APIs in protocol 1, reflecting significant intra-protocol variability when sampling was performed without mixing. Standard deviations exceeded 9% for several APIs, including phenobarbital, clonidine hydrochloride, sildenafil citrate, and furosemide, indicating poor reproducibility of dose recovery under these conditions.

Gentle manual mixing prior to sampling (protocol 2) resulted in higher recovered doses for all APIs compared to protocol 1. The mean recovered doses ranged from 33.83 ± 6.12% for ursodesoxycholic acid to 96.04 ± 4.97% for captopril. The recovered dose for captopril was the same as the target value (96.04 ± 4.97%, *p* = 0.504, Table 3). Recovered doses which were close to the target value but statistically different (*p* < 0.05) were observed for thiamine hydrochloride (94.80 ± 6.20%), and clonidine hydrochloride (88.97 ± 1.74%). Good-to-moderate recovered doses were obtained for sildenafil citrate (77.32 ± 6.55%), amiodarone hydrochloride (57.04 ± 4.75%) and nicardipine hydrochloride (55.45 ± 5.01%). Lower recovered doses persisted for furosemide (47.78 ± 6.30%), spironolactone (42.98 ± 4.19%), and ursodesoxycholic acid (33.83 ± 6.12%). Compared with protocol 1, standard deviations were generally lower in protocol 2, indicating better reproducibility after gentle manual mixing.

When sampling was performed 10 s after mixing (protocol 3), recovered doses were generally similar to or lower than those obtained with protocol 2. The mean recovered doses ranged from 33.32 ± 4.33% for ursodesoxycholic acid to 96.48 ± 4.53% for thiamine hydrochloride. For thiamine hydrochloride and captopril, the recovered doses remained close to the target value but statistically different (*p* < 0.05), with mean contents of 96.48 ± 4.53% and 91.83 ± 2.56%, respectively. Clonidine hydrochloride showed a moderate decrease compared to protocol 2 (76.62 ± 10.09%). For several APIs, including phenobarbital (43.70 ± 7.01%), nicardipine hydrochloride (40.41 ± 11.68%), amiodarone hydrochloride (47.68 ± 4.81%), and furosemide (36.92 ± 8.47%), the recovered doses remained well below the target value. It should be noted that protocols 2 and 3 led to similar recovered doses for clonidine (*p* = 0.978), phenobarbital (*p* = 0.211), thiamine hydrochloride (*p* = 0.770), captopril (*p* = 0.079), spironolactone (*p* = 0.183), and ursodesoxycholic acid (*p* = 0.978) (Table 4).

## 3. Discussion

Our results showed differences between the dispersion protocols tested. Dispersion without systematic mixing led to an underdosing of the API, yielding at best less than 60% of the theoretical content, but generally equal to about 20% of the theoretical content. In practice, capsule dispersion can be performed by nursing staff in hospitals or by parents for outpatients. It is therefore difficult to assume reproducibility between doses administered to the same patient.

If the dispersion is mixed gently and administered immediately, the administered dose increases significantly. APIs with a theoretical content lower than their solubility in water (resulting in a solution) have an administered dose very close to the theoretical content (clonidine hydrochloride, thiamine hydrochloride, captopril, and sildenafil citrate). The slight differences observed could be related to a lack of homogeneity, to an adsorption on the capsule shell or to an agglomerate of insoluble microcrystalline cellulose (the diluent) trapping the active ingredient. In this study, the only active ingredient that delivers a dose equal to the theoretical dose is captopril (*p* = 0.504). Among these, sildenafil citrate is the API with the closest theoretical water solubility (1–10 mg/mL) and theoretical content (1 mg/mL) values, but also the one with the lowest administered dose value (77.32 ± 6.55% with protocol 2). It is worse for nicardipine, where even if the theorical content (0.25 mg·mL^−1^) is four times below the lower limit of solubility (1–10 mg/mL), the recovered dose is only about half of the expected value (55.45 ± 5.01%).

On the other hand, APIs whose theoretical content exceeds their solubility in water (resulting in a suspension) all lead to underdosing (phenobarbital, spironolactone, amiodarone, ursodeoxycholic acid, nicardipine hydrochloride, furosemide). This phenomenon is accentuated when the dispersion is left to stand for 10 s before sampling (protocol 3), strongly suggesting API sedimentation. Unfortunately, as insoluble microcrystalline cellulose was used as a diluent during capsule formulation, we cannot objectivate this assertion.

However, even though the analyses were performed just after the dispersion of the capsules in water, we cannot exclude an API degradation, which will increase the difference between the theoretical versus the real-administered dose. This is particularly true for light-sensitive APIs such as furosemide [12] or nicardipine [13] or for hydrolysable and oxidizable APIs such as captopril [14].

These results allow us to make the following recommendations:

- Whenever possible, always prepare capsules whose contents correspond to the prescribed dose.
- If capsules are prepared in advance and stored, and if the prescribed dose does not correspond to the contents of the capsules, an oral liquid formulation should be preferred over dispersing the capsules in water and fractionated sampling prior to administration.
- If a liquid formulation is not possible, a pharmaceutical expertise to determine whether the dispersed capsule will result in a solution or a suspension should be mandatory.

In pediatric compounding practice, the safety of the formulation must always be prioritized. Therefore, special attention is paid, for example, to the presence of any excipient with noticeable effects (such as preservatives) and to the osmolarity of the formula. Hard gelatin capsules with only one excipient (diluent) appear as a good alternative, and oral medication standardization has proven its benefit on improving pediatric outcomes [15]. However, our results show that, under hospital compounding policy, the risk–benefit balance between a liquid and a dry oral formulation must always be evaluated by a compounding pharmacist. Moreover, as pediatric safety is clearly identified as a hot topic [16,17], the compounding practice remains under-evaluated despite its implication in several dramatic drug medication errors [18].

Our work presents limitations as we only evaluated protocol with fractioned sampling (capsule dispersed in 2 mL, and only 1 mL is administered), for example when the dose prescribed is “ursodesoxycholic acid 10 mg” and there are only 20 mg compounded capsules stored at the pharmacy. However, it would be interesting to evaluate the administered dose when all the water volume is administered. It is possible that a suspension quickly sediments, but also that the API remains adsorbed on the surface of the container. To evaluate this potential phenomenon, additional investigations are currently in progress.

Moreover, we only evaluated one sample preparation method, with a fixed volume of 2 mL, which is suggested as the highest volume of liquid drugs to be orally administered to an infant or toddler [11]. However, larger volumes of water could have been evaluated. Nevertheless, even though the addition of a natural surfactant in the water to improve drug solubility or thickening agents to improve homogeneity could also have been studied, we focused on the constraints of clinical practice. Thus, such substances will increase the osmolarity of the resulting mixture while this parameter must be as low as possible in pediatric practice (lower than 450 mOsm.kg^−1^) to minimize the risk of necrotizing enterocolitis [19].

Several factors have an influence on the sedimentation process, such as the temperature or the density and the charge and the size of suspended particles [20]. Among these, an increase in particle concentration, correlated to the ratio of the “number of particles” to “volume”, leads to a viscosity increase [21]. Therefore, it could be hypothesized that a low dispersion volume such as 2 mL for poorly soluble APIs (which were not soluble whatever the volume) will result in a better suspension homogeneity and the best accuracy on the recovery dose. However, as the sedimentation kinetics vary though a wide variety of factors [22,23,24,25], it is difficult to conclude a clear and linear relation between the dispersion volume and the accuracy of the administered dose.

## 4. Material and Methods

Each API was quantified with an in-house validated HPLC-UV method routinely used to control compounded capsules for hospitalized patients. Several have already been described in the literature by our group: clonidine [26], thiamine hydrochloride [27], captopril [28], spironolactone [29], amiodarone hydrochloride [30], and nicardipine hydrochloride [29].

All the chromatographic methods were performed on an automatic HPLC-UV-DAD system Dionex UltiMate 3000 HPLC System (Thermo Fisher Scientific, Waltham, MA, USA) with software Chromeleon 7.2.8. (Thermo Fisher Scientific, Waltham, MA, USA).

### 4.1. Study Design

First, for each drug, a batch of 100 size 4 capsules (LGA, La Seyne-sur-Mer, France) was produced manually with a nonautomated capsule filling machine (Cooper, Melun, France) to reflect small-scale compounding, which is the most frequent practice in hospital and ambulatory pharmacies. API and excipients were weighted with a Precisa XT 220 A balance (Precisa, Poissy, France). Batches were stored at ambient temperature in a 100 mL opaque plastic jar closed with a screw cap (Cooper, Melun, France) for no more than two weeks before analysis. These batches were only produced for experimental purposes and were not administered to patients. Ten individual capsules were analyzed to determine the content uniformity (using the appropriate test from the European Pharmacopoeia, 2.9.6. or 2.9.40., depending on the theoretical content), and batch content was defined as the mean value of the content of 10 individual capsules.

In order to assess the actual amount of medication administered to the patient when a partial volume is withdrawn, three different protocols were implemented to simulate what nurses might do. At the start of all protocols, one capsule of the API under study was dispersed in 2 mL of ultrapure water. Capsules were opened and their content poured in a 7 mL standard square polystyrene weighing boat—Heathrow Scientific HS1420A (Vernon Hills, IL, USA). In protocol 1, 1 mL of the supernatant was aliquoted using an insulin syringe and diluted in order to analyze the drug content. In protocol 2, the water/drug mixture was carefully mixed manually three times, then 1 mL of the supernatant was immediately withdrawn using an insulin syringe and diluted to analyze the drug content. In protocol 3, the water/drug mixture was carefully mixed manually three times, then 1 mL of the supernatant was taken after 10 s of rest using an insulin syringe and diluted for analysis of the drug content.

The analyses were performed just after the capsule dispersion in water to minimize API degradation.

For each drug and each protocol, the dosage was performed 10 times (*n* = 10). The results were expressed as mean content +/− standard deviation.

### 4.2. Chemicals and Reagents

Type 1 Water (Ultrapure Water) was obtained using the Merck Millipore Direct-Q^®^ 3 UV (Merck KGaA, Darmstadt, Germany) water purification system.

Active Pharmaceutical Ingredients were purchased from INRESA (Barthenheim, France) for clonidine (batch N° 2202002451), phenobarbital (batch N° 2024.02.0481), captopril (batch N° 5102-23-017), sildenafil (batch N° SF/1/21019/00A), spironolactone (batch N° 2108523210), amiodarone (batch N° 22AM000056), ursodeoxycholic acid (batch N° F2200172), nicardipine (batch N° 21500 00940) and furosemide (batch N° 008534). Thiamine (batch N° 22110223A) was purchased from COOPER (Melun, France). Excipients for capsules were microcrystalline cellulose (COOPER, batch N° 22110011/C) and cochineal red A (E124) (FAGRON, Rotterdam, The Netherlands) (batch N° 224112).

### 4.3. Chromatographic Conditions for the Dosage of Furosemide

The chromatographic column was XTerra^®^ RP18 5 µm, 4.6 × 250 mm column (Waters, Milford, MA, USA). The mobile phase was prepared as follows:

In a 2 L beaker, 700 mL of UP water was added and buffered with 1 mL of glacial acetic acid. Then, 300 mL of HPLC-grade acetonitrile was added. The mobile phase was filtered through a 0.45 µm filter and sonicated for 15 min before being used.

Isocratic mode was used at a flow of 1.5 mL.min^−1^ for 20 min. The wavelength detection was 272 nm, injection volumes were 20 µL and the column temperature was 22 °C. The furosemide retention time was 14 min.

The following parameters were evaluated in the method validation from a furosemide standard (furosemide Certified Reference Material, PHR1057, Sigma-Aldrich, St Louis, MO, USA): linearity, repeatability (within-day variation), intermediate precision (between-day variation) and accuracy. Within-day and between-day measurements were performed at three concentrations (180, 200 and 220 µg·mL^−1^). Linearity was investigated with five concentrations ranging from 50 to 800 µg·mL^−1^ prepared in sextuplicate and was demonstrated between 50 and 800 µg·mL^−1^. The results are summarized in Table S1 (Supplementary Materials).

Capsule samples were treated as follows: 2 mg furosemide capsules were opened and the content was poured into a 20 mL plastic centrifuge tube. Next, 10 mL of mobile phase was added, vortexed for 5 min, then centrifuged for 5 min at 3000× *g* rpm. The supernatant was filtered before transferring 200 µL of the resulting solution to a vial. For each analysis, two independent quality controls (QC1 and QC2) were performed using furosemide European Pharmacopoeia (EP) Primary Standard (F0700000, Sigma-Aldrich, St Louis, MO, USA). QC1 was analyzed once and QC2 was analyzed six times. QC1 and QC2 were used to quantify the furosemide capsule content and for the System Suitability Test.

### 4.4. Chromatographic Conditions for the Dosage of Phenobarbital

The chromatographic column was XTerra^®^ RP18 5 µm, 4.6 × 250 mm column (Waters, Milford, MA, USA). The mobile phase is prepared as follows: In a 1 L bottle, pour 800 mL of UP water, then add 200 mL of ACN. Adjust the pH to exactly 3.00 using 0.178 M diluted sulfuric acid prepared from 95% sulfuric acid. It was filtered through a Millipore 0.45 µm cellulose filter (Burlington, MA, USA) and used in isocratic mode at a flow of 1 mL.min^−1^ for 15 min. The wavelength detection was 235 nm, injection volumes were 5 µL and the column temperature was 60 °C. The phenobarbital retention time was 11.2 min. The following parameters were evaluated in the method validation from a phenobarbital standard (phenobarbital certified reference material, PHR8843-500MG, Sigma-Aldrich, St Louis, MO, USA): linearity, repeatability (within-day variation), intermediate precision (between-day variation) and accuracy. Within-day and between-day measurements were performed at three concentrations (0.45, 0.50 and 0.55 mg·mL^−1^). Linearity was investigated with seven concentrations ranging from 0.05 to 1 mg·L^−1^ prepared in septuplicate and was demonstrated between 0.05 and 1 mg·L^−1^. The results are summarized in Table S1 (Supplementary Materials).

Capsule samples were treated as follows: 20 mg phenobarbital capsules were opened and poured with the content into a 50 mL plastic centrifuge tube. Next, 40 mL of mobile phase was added, vortexed for 10 min, then centrifuged for 5 min at 3000× *g* rpm. The supernatant was filtered before transferring 200 µL of the resulting solution to a vial. For each analysis, two independent quality controls (QC1 and QC2) were performed using phenobarbital European Pharmacopoeia (EP) Primary Standard (Sigma-Aldrich, St Louis, MO, USA). QC1 was analyzed once and QC2 was analyzed six times. QC1 and QC2 were used to quantify the phenobarbital capsule content and for the System Suitability Test.

### 4.5. Chromatographic Conditions for the Dosage of Sildenafil

The chromatographic column was Inertsil C18, 5 µm, 150 mm X 4.6 mm column (GL Sciences Inc., Tokyo, Japan). The mobile phase was prepared as follows:

In a 500 mL beaker, 0.207 g of sodium phosphate monohydrate was added, then 300 mL of UP water. The pH was adjusted to 7 with triethylamine. The aqueous phase was filtered using the 500 mL vacuum flask fitted with a 0.45 µm U-PVDF filter. Then, 700 mL of ACN was added and sonicated for 15 min.

Isocratic mode was used at a flow of 0.8 mL.min^−1^ for 6 min. The wavelength detection was 228 nm, injection volumes were 10 µL and the column temperature was 22 °C. The sildenafil retention time was 3.4 min.

The following parameters were evaluated in the method validation from sildenafil citrate standard (sildenafil citrate Certified Reference Material, PHR1807, Sigma-Aldrich, St Louis, MO, USA): linearity, repeatability (within-day variation), intermediate precision (between-day variation) and accuracy. Within-day and between-day measurements were performed at three concentrations (35, 70 and 80 µg.mL^−1^). Linearity was investigated with five concentrations ranging from 6.25 to 100 µg.L^−1^ prepared in sextuplicate and was demonstrated between 6.25 and 100 µg.L^−1^. The results are summarized in Table S1 (Supplementary Materials).

Capsule samples were treated as follows: 2 mg sildenafil citrate capsules were opened and the content was poured into a 50 mL plastic centrifuge tube. Next, 25 mL of mobile phase was added, vortexed for 5 min, then centrifuged for 5 min at 3000× *g* rpm. The supernatant was filtered before transferring 200 µL of the resulting solution to a vial. For each analysis, two independent quality controls (QC1 and QC2) were performed using sildenafil European Pharmacopoeia (EP) Primary Standard (Y0001578, Sigma-Aldrich, St Louis, MO, USA). QC1 was analyzed once and QC2 was analyzed six times. QC1 and QC2 were used to quantify the sildenafil capsule content and for the System Suitability Test.

### 4.6. Chromatographic Conditions for the Dosage of Ursodeoxycholic Acid

The chromatographic column was XTerra^®^ RP18 5 µm, 4.6 × 250 mm column (Waters, Milford, MA, USA). The mobile phase was prepared as follows:

In a 1 L beaker, 0.408 g of monopotassium phosphate was added and dissolved in 300 mL of UP water (10 mM phosphate buffer). The solution was stirred magnetically until completely dissolved. The pH was adjusted to 2.5 with orthophosphoric acid. Next, 700 mL of HPLC-grade methanol was added. The mobile phase was filtered through a 0.45 µm filter and sonicated for 15 min before being used.

Isocratic mode was used at a flow of 0.8 mL.min^−1^ for 12 min. The wavelength detection was 200 nm, injection volumes were 75 µL and the column temperature was 60 °C. The ursodeoxycholic acid retention time was 7 min.

The following parameters were evaluated in the method validation from ursodeoxycholic acid standard (ursodeoxycholic acid Certified Reference Material, PHR1579, Sigma-Aldrich, St Louis, MO, USA): linearity, repeatability (within-day variation), intermediate precision (between-day variation) and accuracy. Within-day and between-day measurements were performed at three concentrations (100, 300 and 330 µg.mL^−1^). Linearity was investigated with six concentrations ranging from 50 to 500 µg·L^−1^ prepared in sextuplicate and was demonstrated between 100 and 500 µg·L^−1^. The results are summarized in Table S1 (Supplementary Materials).

Capsule samples were treated as follows: 20 mg ursodeoxycholic acid capsules were opened and poured with the content into a 50 mL plastic centrifuge tube. Next, 10 mL of mobile phase was added, vortexed for 5 min, then centrifuged for 7 min at 2000× *g* rpm. 250 µL of supernatant was pipetted, poured into a vial and 850 µL of mobile phase was added and then homogenized. For each analysis, two independent quality controls (QC1 and QC2) were performed using ursodeoxycholic acid European Pharmacopoeia (EP) Primary Standard (U0800000, Sigma-Aldrich, St Louis, MO, USA). QC1 was analyzed once and QC2 was analyzed six times. QC1 and QC2 were used to quantify the ursodeoxycholic acid capsule content and for the System Suitability Test.

### 4.7. Statistical Analysis

Statistical analyses were performed using IBM SPSS 20 Statistics (IBM Corp., Armonk, NY, USA) software. A *p*-value less than 0.05 was considered statistically significant.

We first investigated whether or not the percentage of recovered drug showed a statistically significant difference from the theoretical content (content uniformity) obtained with the three protocols used (*n* = 10, H_0_ hypothesis: no difference). A normality analysis of the data was performed using the Shapiro–Wilk test. If the data followed a normal distribution, Student’s *t*-test was performed; otherwise, the Wilcoxon signed-rank test was used.

Then, to compare the protocols with each other and determine whether they yielded the same active ingredient content for each API considered, a Shapiro–Wilk test was performed beforehand to show the normality (*p* > 0.05, acceptable normality). A one-way analysis of variance (ANOVA) was performed to compare the variable% of recovered drug content between the three protocols (*n* = 10) of each API. Levene’s test was used to indicate whether or not there was heterogeneity of variances. If the variances were homogeneous, Tukey’s post hoc test was used; otherwise, Games–Howell’s post hoc test was used.

## 5. Conclusions

Solid oral formulations such as compounded capsules offer several advantages in pediatrics (stability, simplicity, safety). Their use requires a preliminary pharmaceutical analysis to determine their feasibility. When capsules are compounded and stored in advance, and if the prescribed dose is not a multiple of the capsule’s contents, liquid oral formulations must be preferred. Furthermore, the dispersion in water of a water-insoluble active ingredient through fractionated administration must be strictly avoided to prevent underdosing.

## Figures and Tables

**Table 1:** Solubility of studied drugs and theoretical content in 2 mL of water.

Hard Gelatin Capsule Analyzed	Theoretical Content	Solubility in Water in European Pharmacopoiea (EP)	Corresponding Water Solubility (EP)
Clonidine hydrochloride 20 µg	0.01 mg·mL^−1^	Soluble	33.3–100 mg·mL^−1^
Phenobarbital 20 mg	10 mg·mL^−1^	Very slightly soluble	0.1–1 mg·mL^−1^
Thiamine hydrochloride 50 mg	25 mg·mL^−1^	Freely soluble	100–1 000 mg·mL^−1^
Captopril 3 mg	1.5 mg·mL^−1^	Soluble	33.3–100 mg·mL^−1^
Sildenafil citrate 2 mg	1 mg·mL^−1^	Slightly soluble	1–10 mg·mL^−1^
Spironolactone 2.5 mg	1.25 mg·mL^−1^	Practically insoluble	<0.1 mg·mL^−1^
Amiodarone hydrochloride 5 mg	2.5 mg·mL^−1^	Very slightly soluble	0.1–1 mg·mL^−1^
Ursodesoxycholic acid 20 mg	10 mg·mL^−1^	Practically insoluble	< 0.1 mg·mL^−1^
Nicardipine hydrochloride 0.5 mg	0.25 mg·mL^−1^	Slightly soluble	1–10 mg·mL^−1^
Furosemide 2 mg	1 mg·mL^−1^	Practically insoluble	<0.1 mg·mL^−1^

**Table 2:** Content uniformity of hard gelatin capsules and drug content recovered according to protocols 1, 2 and 3.

	Content Uniformity (%)	Protocol 1 (%)	Protocol 2 (%)	Protocol 3 (%)
Clonidine hydrochloride 20 µg	93.55 ± 3.16	31.36 ± 11.06	88.97 ± 1.74	76.62 ± 10.09
Phenobarbital 20 mg	95.30 ± 1.37	17.18 ± 11.88	49.62 ± 7.97	43.70 ± 7.01
Thiamine hydrochloride 50 mg	107.68 ± 5.84	57.66 ± 12.08	94.80 ± 6.20	96.48 ± 4.53
Captopril 3 mg	97.13 ± 4.81	41.40 ± 10.72	96.04 ± 4.97	91.83 ± 2.56
Sildenafil citrate 2 mg	99.28 ± 4.81	39.64 ± 11.62	77.32 ± 6.55	60.43 ± 6.47
Spironolactone 2.5 mg	94.76 ± 5.01	20.94 ± 6.14	42.98 ± 4.19	38.62 ± 5.57
Amiodarone hydrochloride 5 mg	102.25 ± 5.08	25.11 ± 8.30	57.04 ± 4.75	47.68 ± 4.81
Ursodesoxycholic acid 20 mg	100.41 ± 4.52	21.16 ± 6.48	33.83 ± 6.12	33.32 ± 4.33
Nicardipine hydrochloride 0.5 mg	103.02 ± 4.65	22.77 ± 8.81	55.45 ± 5.01	40.41 ± 11.68
Furosemide 2 mg	95.14 ± 3.95	20.87 ± 9.04	47.78 ± 6.30	36.92 ± 8.47

**Table 3:** Tests for differences in means between content of uniformity and protocol 1 (P1), protocol 2 (P2) and protocol 3 (P3) for each API (*n* = 10).

	Mean Difference	95% Confidence Interval of the Difference	*p*-Value
Clonidine P1	−62.194	(−70.107, −54.281)	0.000 ^a^
Clonidine P2	−12.154	(−18.497, −5.811)	0.000 ^a^
Clonidine P3	0.512	(−5.831, 6.855)	0.000 ^a^
Phenobarbital P1	−78.120	(−86.622, −69.618)	0.000 ^b^
Phenobarbital P2	−45.680	(−51.385, −39.975)	0.000 ^a^
Phenobarbital P3	−51.600	(−56.615, −46.585)	0.000 ^a^
Thiamine P1	−50.024	(−58.664, −41.384)	0.000 ^a^
Thiamine P2	−12.884	(−17.317, 8.451)	0.000 ^a^
Thiamine P3	−11.200	(−14.438, −7.962)	0.000 ^a^
Captopril P1	−55.725	(−63.390, −48.060)	0.000 ^a^
**Captopril P2**	**−1.094**	**(4.649, 2.462)**	**0.504 ^b^**
Captopril P3	−5.296	(−7.128, −3.464)	0.000 ^a^
Sildenafil P1	−59.644	(−67.956, −51.331)	0.000 ^a^
Sildenafil P2	−21.964	(−26.647, −17.281)	0.000 ^a^
Sildenafil P3	−38.849	(−43.478, −34.220)	0.000 ^a^
Spironolactone P1	−73.822	(−78.212, −69.432)	0.000 ^a^
Spironolactone P2	−51.784	(−54.784, −48.784)	0.000 ^a^
Spironolactone P3	−56.139	(−60.123, −52.155)	0.000 ^a^
Amiodarone P1	−77.139	(−83.075, −71.203)	0.000 ^a^
Amiodarone P2	−45.210	(−48.609, −41.811)	0.000 ^a^
Amiodarone P3	−54.568	(58.009, −51.127)	0.000 ^a^
Ursodesoxycholic acid P1	−79.245	(−83.882, −74.608)	0.000 ^a^
Ursodesoxycholic acid P2	−66.579	(−70.955, −62.203)	0.000 ^a^
Ursodesoxycholic acid P3	−67.091	(−70.186, −63.996)	0.000 ^a^
Nicardipine P1	−80.253	(−86.554, −73.952)	0.000 ^a^
Nicardipine P2	−47.563	(−51.149, 43.977)	0.000 ^a^
Nicardipine P3	−62.614	(−70.970, −54.258)	0.000 ^a^
Furosemide P1	−74.271	(−80.738, −67.804)	0.000 ^a^
Furosemide P2	−47.358	(−51.867, −42.849)	0.000 ^a^
Furosemide P3	−58.221	(−64.280, −52.163)	0.000 ^a^

^a^ Student’s *t*-test was used (Shapiro test of normality > 0.05). ^b^ Wilcoxon signed-rank test was used (Shapiro test of normality < 0.05) and the conclusion was the same as Student’s *t*-test; the reported *p*-value is Student’s *t*-test. Bold was used to highlight significant *p*-value.

**Table 4:** ANOVA tests for difference in means between protocol 1 (P1), protocol 2 (P2) and protocol 3 (P3) for each API (*n* = 10).

	Mean Difference	Standard Error of Mean Difference	95% Confidence Interval of the Difference	*p*-Value
Clonidine P1–P2	−12.666	2.558	(−19.009, −6.323)	0.000 ^a^
Clonidine P1–P3	−12.154	2.558	(−18.497, −5.811)	0.000 ^a^
**Clonidine P2–P3**	**0.512**	**2.558**	**(−5.831, 6.855**)	0.978 *^a^
Phenobarbital P1–P2	−32.440	4.526	(−44.138, −20.742)	0.000 ^b^
Phenobarbital P1–P3	−26.520	4.363	(−37.888, −15.152)	0.000 ^b^
**Phenobarbital P2–P3**	**5.920**	**3.357**	**(−2.662, 14.502)**	0.211 *^b^
Thiamine P1–P2	−37.140	4.293	(−48,429, −25,851)	0.000 ^b^
Thiamine P1–P3	−38.824	4.079	(−49.772, −27,876)	0.000 ^b^
**Thiamine P2–P3**	**−1.684**	**2.427**	**(−7.928, 4.560)**	0.770 *^b^
Captopril P1–P2	−54.631	3.735	(−64.523, −44.740)	0.000 ^b^
Captopril P1–P3	−50.429	3.484	(−59.976, −40.883)	0.000 ^b^
**Captopril P2–P3**	**4.202**	**1.768**	**(−0.447, 8.851)**	0.079 *^b^
Sildenafil P1–P2	−37.680	3.828	(−47.170, −28,190)	0.000 ^a^
Sildenafil P1–P3	−20.795	3.828	(−30.285, −11.305)	0.000 ^a^
Sildenafil P2–P3	16.885	3.828	(7.395, 26.375)	0.000 ^a^
Spironolactone P1–P2	−22.038	2.398	(−27.984, −16.092)	0.000 ^a^
Spironolactone P1–P3	−17.683	2.398	(−23.629, −11.737)	0.000 ^a^
**Spironolactone P2–P3**	**4.355**	**2.398**	**(−1.591, 10.301)**	0.183 *^a^
Amiodarone P1–P2	−31.929	2.764	(−38.781, −25.077)	0.000 ^a^
Amiodarone P1–P3	−22.571	2.764	(−29.423, −15.719)	0.000 ^a^
Amiodarone P2–P3	9.358	2.764	(2.506, 16.210)	0.006 ^a^
Ursodesoxycholic acid P1–P2	−12.666	2.558	(−19.009, −6.323)	0.000 ^a^
Ursodesoxycholic acid P1–P3	−12.154	2.558	(−18.497, −5.811)	0.000 ^a^
**Ursodesoxycholic acid P2–P3**	**0.512**	**2.558**	**(−5.831, 6.855)**	0.978 *^a^
Nicardipine P1–P2	−32.690	3.204	(−41.059, −24.321)	0.000 ^b^
Nicardipine P1–P3	−17.639	4.626	(−29.525, −5.753)	0.004 ^b^
Nicardipine P2–P3	15.051	4.020	(4.351, 25.751)	0.007 ^b^
Furosemide P1–P2	−26.913	3.589	(−35.811, −18.015)	0.000 ^a^
Furosemide P1–P3	−16.050	3.589	(24.948, −7.152)	0.000 ^a^
Furosemide P2–P3	10.863	3.589	(1.965, 19.761)	0.014 ^a^

^a^ Tukey’s test was used as a post hoc test (homogeneity of variances > 0.05). ^b^ Games–Howell was used as a post hoc test (homogeneity of variances < 0.05). * *p* > 0.05, indicates that no statistical difference was found between the protocols. Bold was used to highlight significant *p*-value.

## Data Availability

The original contributions presented in this study are included in the article/Supplementary Material. Further inquiries can be directed to the corresponding author.

## Supplementary Materials

The following supporting information can be downloaded at https://www.mdpi.com/article/10.3390/ph19040534/s1: Figure S1. Content uniformity of clonidine hydrochloride 20 µg and recovered drug content according to protocols 1, 2 and 3; Figure S2. Content uniformity of phenobarbital 20 mg and recovered drug content according to protocols 1, 2 and 3; Figure S3. Content uniformity of thiamine hydrochloride 50 mg and recovered drug content according to protocols 1, 2 and 3; Figure S4. Content uniformity of captopril 3 mg and recovered drug content according to protocols 1, 2 and 3; Figure S5. Content uniformity of sildenafil citrate 2 mg and recovered drug content according to protocols 1, 2 and 3; Figure S6. Content uniformity of spironolactone 2.5 mg and recovered drug content according to protocols 1, 2 and 3; Figure S7. Content uniformity of amiodarone hydrochloride 5 mg and recovered drug content according to protocols 1, 2 and 3; Figure S8. Content uniformity of ursodesoxycholic acid 20 mg and recovered drug content according to protocols 1, 2 and 3; Figure S9. Content uniformity of nicardipine hydrochloride 0.5 mg and recovered drug content according to protocols 1, 2 and 3; Figure S10. Content uniformity of Furosemide 2 mg and recovered drug content according to protocols 1, 2 and 3; Table S1. Repeatability, intermediate precision and accuracy for furosemide, phenobarbital, sildenafil and ursodeoxycholic acid.
