# Supplementary material for: Hard Gelatin Capsules Compounded and Dispersed in Water in Pediatrics: Real Versus Theoretical Dose Administered
Source: Pharmaceuticals (Basel). 2026 Mar 25;19(4):534. doi: 10.3390/ph19040534 (PMC13118315; doi:10.3390/ph19040534)
Supplement: Supplementary file 1 [file pharmaceuticals-19-00534-s001.zip › pharmaceuticals-4185278-supplementary.pdf]

Hard gelatin capsules compounded and dispersed in water in pediatrics: real versus theoretical dose administered

Romain Paoli-Lombardo,[1-2] Nicolas Primas,[1-2] Clémence Tabélé,[3] Ikram Zaddam,[1] Eya Iben Slimene,[1] Pascal Rathelot,[1-2] Patrice Vanelle,[1-2] Caroline Castera-Ducros,[1-2] Christophe Curti.[1-2]

1 Service Central de la Qualité et de l'Information Pharmaceutiques (SCQIP), Pharmacy Department, AP-HM, 174 Boulevard Baille, 13005 Marseille, France.

2 Aix Marseille Université, CNRS, Institut de Chimie Radicalaire ICR, UMR 7273, Equipe Pharmaco-Chimie Radicalaire, 27 Boulevard Jean Moulin, 13385 Marseille, France.

3 Health Services and Quality of Life Research, Aix-Marseille University, School of Medicine, 27 Boulevard Jean Moulin, 13385 Marseille, France.

Figure S1. Content uniformity of clonidine hydrochloride 20 µg and recovered drug content according to protocols 1, 2 and 3

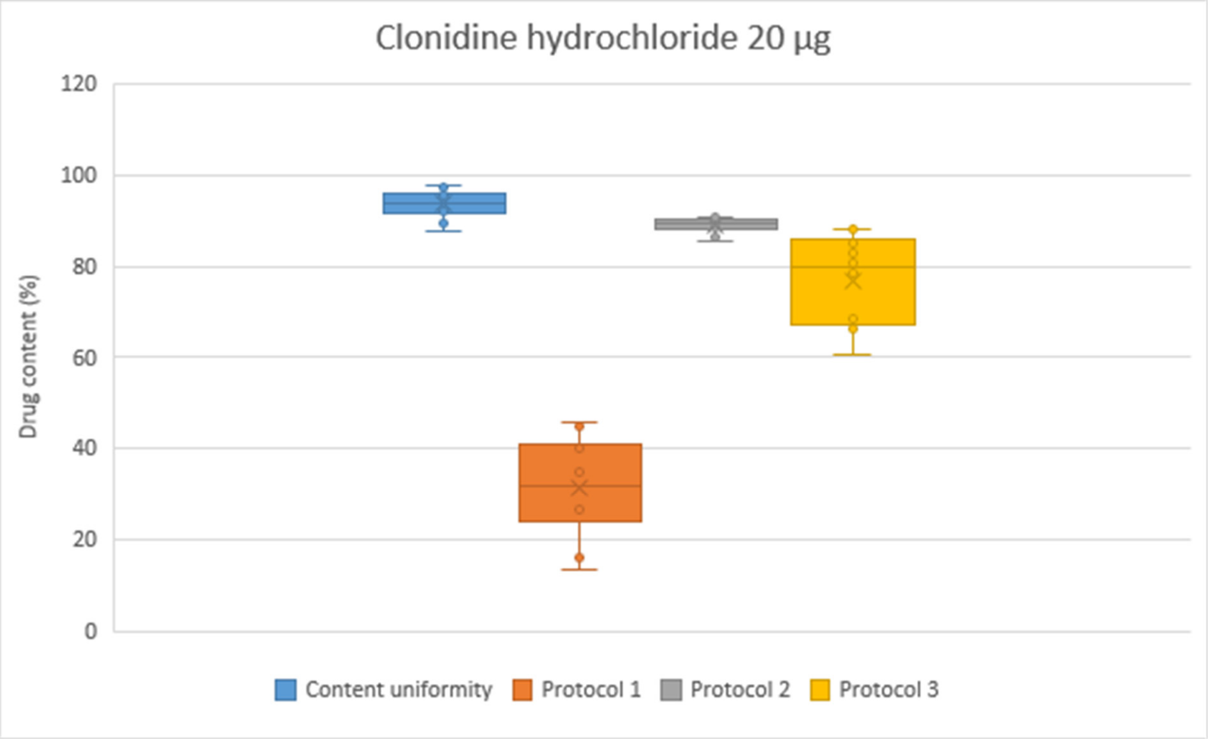

Figure S2. Content uniformity of phenobarbital 20 mg and recovered drug content according to protocols 1, 2 and 3

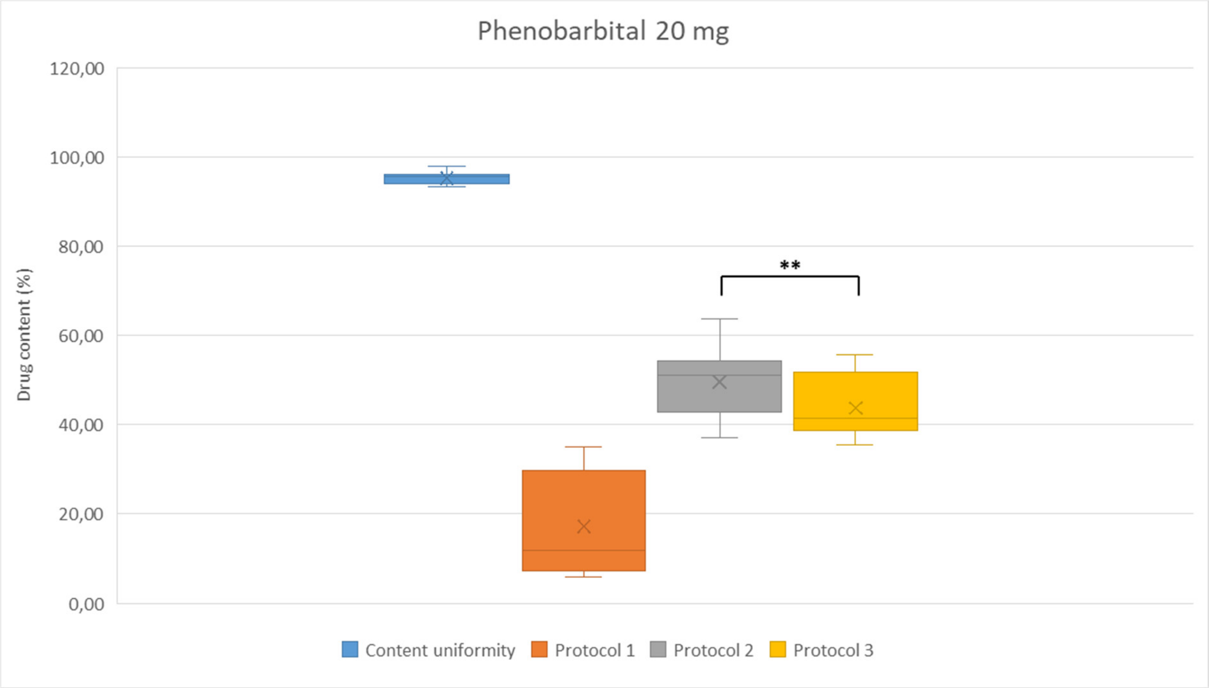

** : ANOVA, $p > 0.05$

Figure S3. Content uniformity of thiamine hydrochloride 50 mg and recovered drug content according to protocols 1, 2 and 3

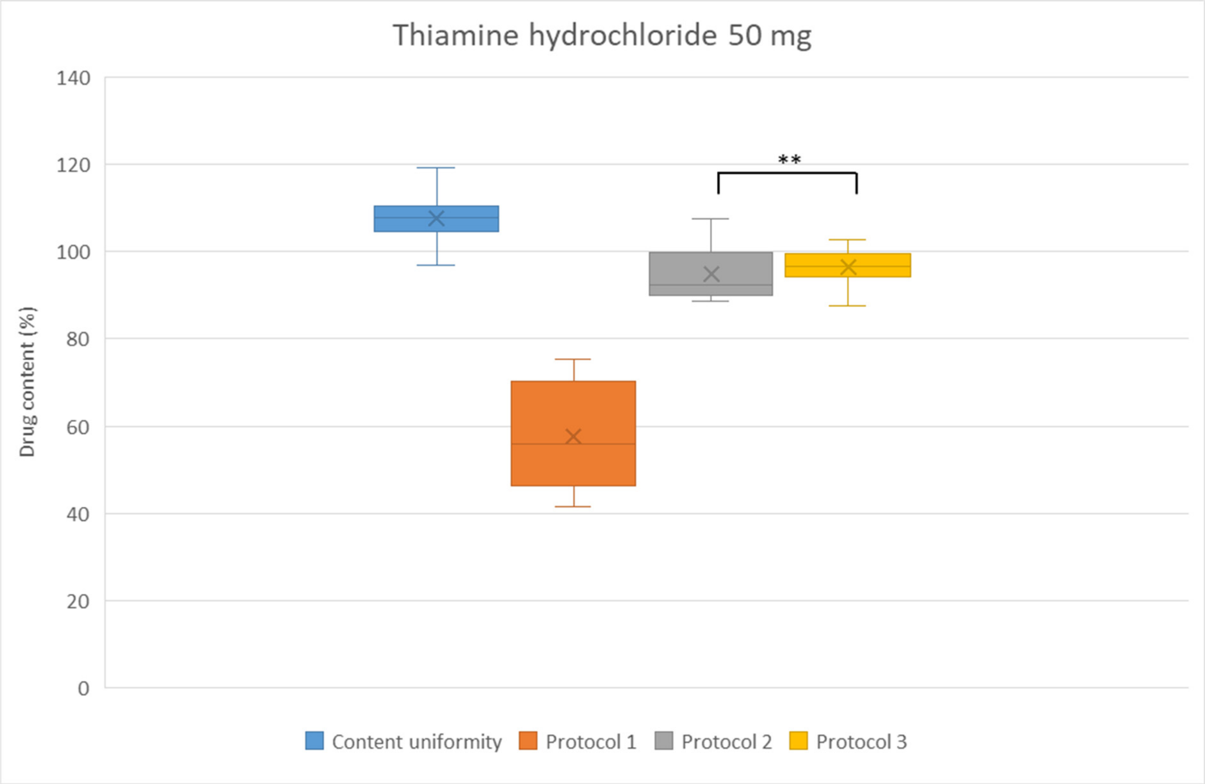

** : ANOVA, $p > 0.05$

Figure S4. Content uniformity of captopril 3 mg and recovered drug content according to protocols 1, 2 and 3

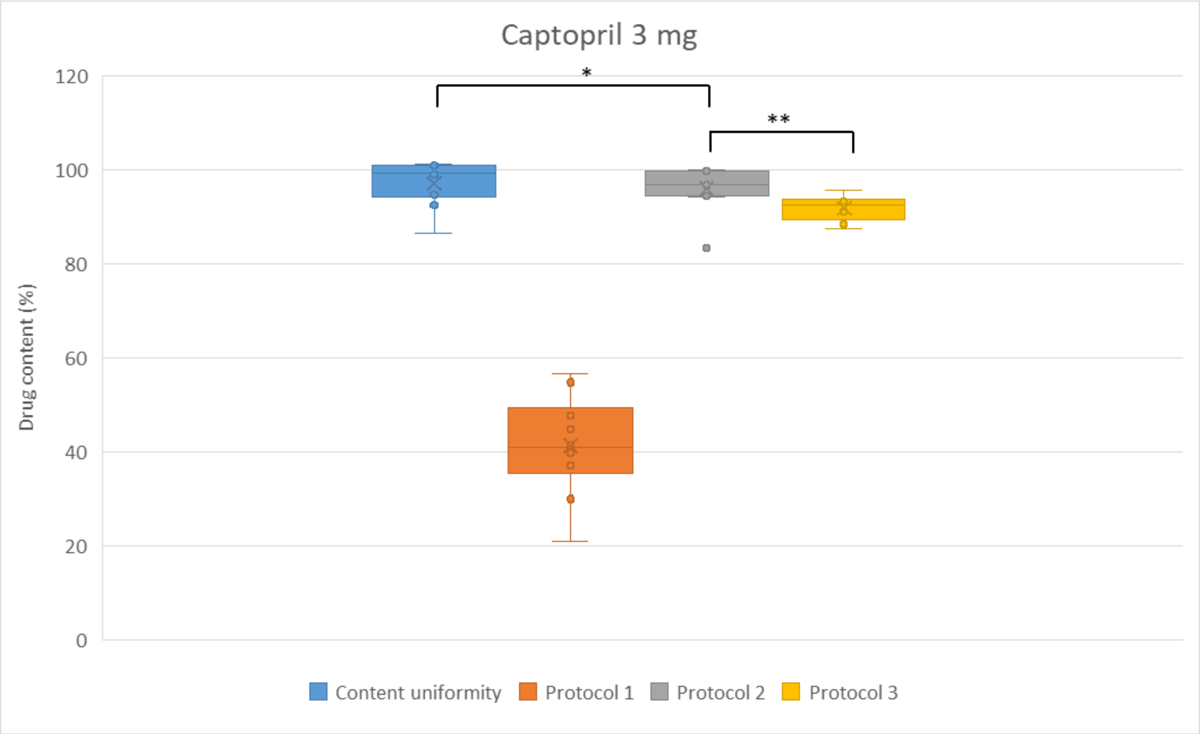

* : Wilcoxon signed rank test, $p > 0.05$
** : ANOVA, $p > 0.05$

Figure S5. Content uniformity of sildenafil citrate 2 mg and recovered drug content according to protocols 1, 2 and 3

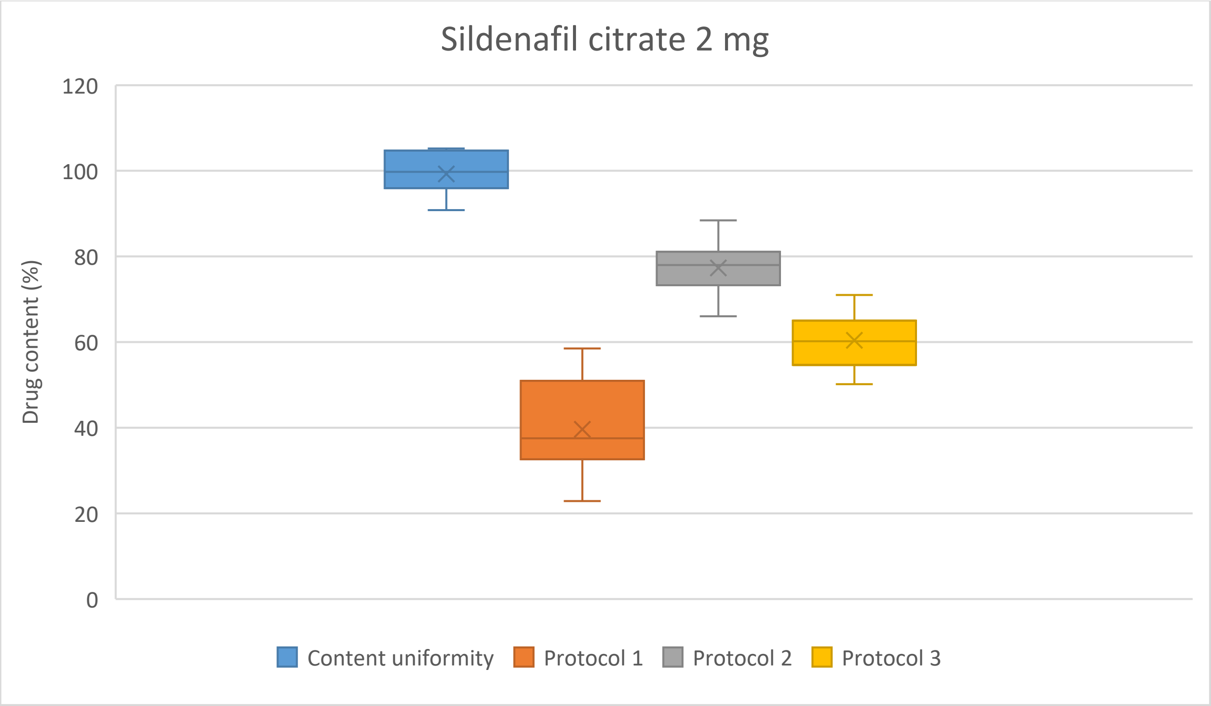

Figure S6. Content uniformity of spironolactone 2.5 mg and recovered drug content according to protocols 1, 2 and 3

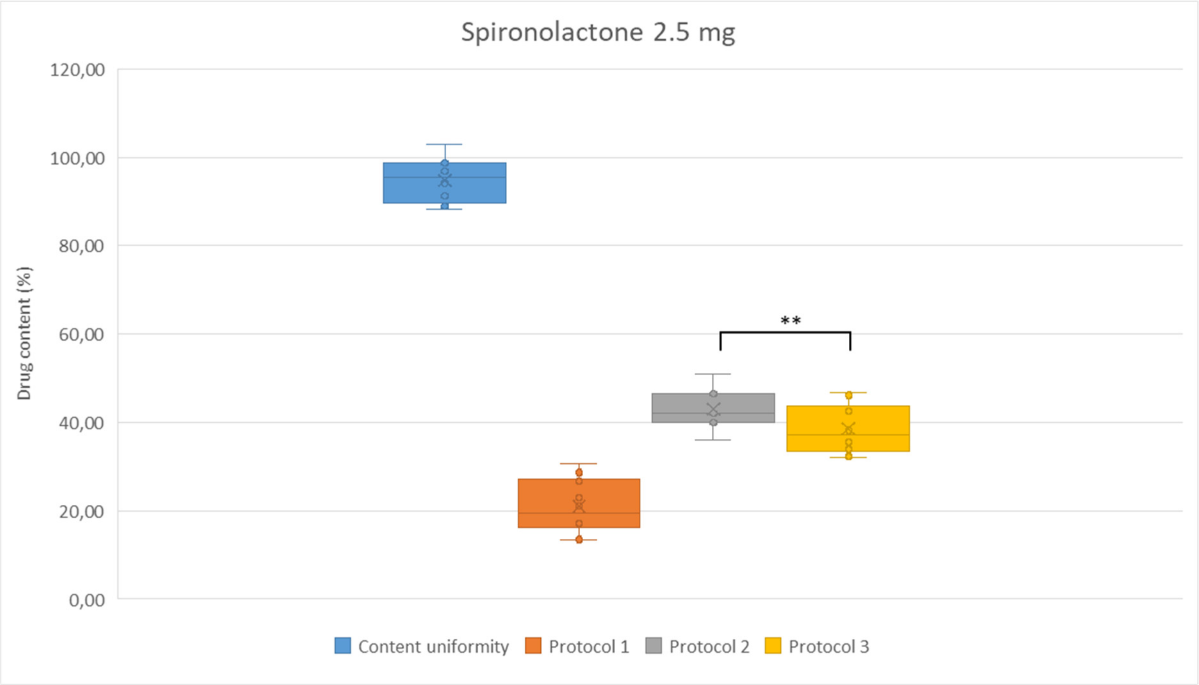

** : ANOVA, $p > 0.05$

Figure S7. Content uniformity of amiodarone hydrochloride 5 mg and recovered drug content according to protocols 1, 2 and 3

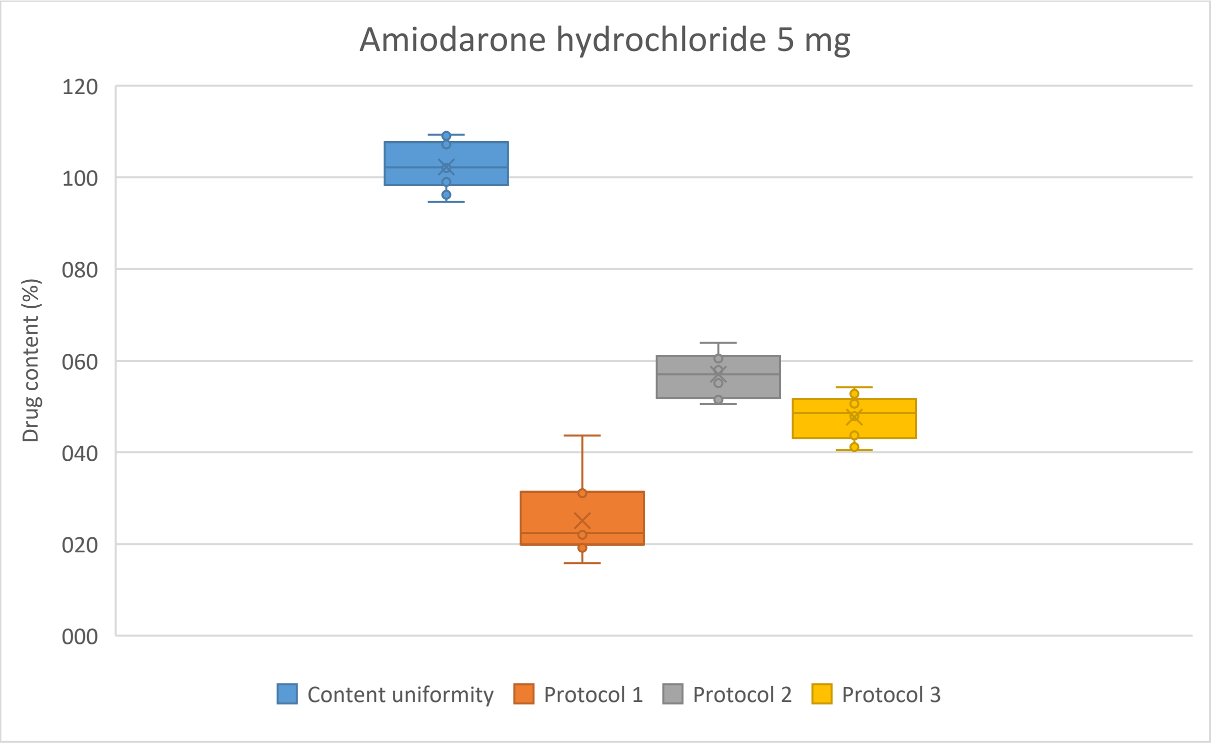

Figure S8. Content uniformity of ursodesoxycholic acid 20 mg and recovered drug content according to protocols 1, 2 and 3

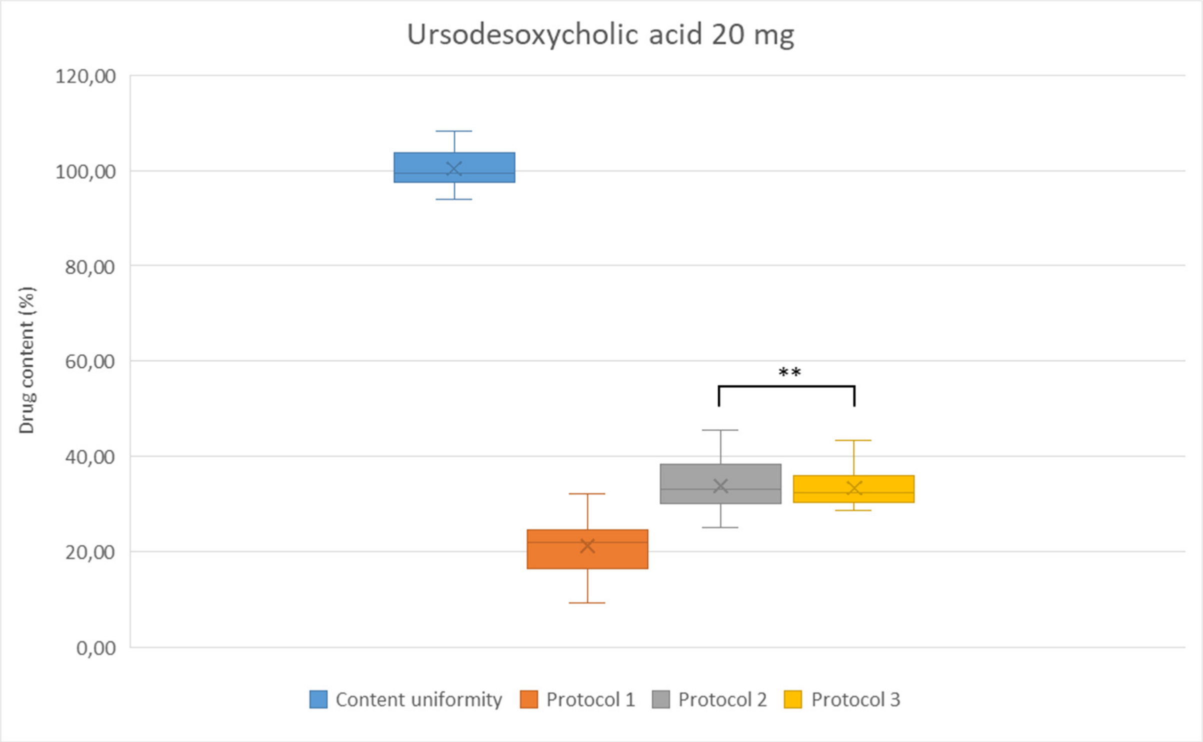

** : ANOVA, *p* > 0.05

Figure S9. Content uniformity of nicardipine hydrochloride 0.5 mg and recovered drug content according to protocols 1, 2 and 3

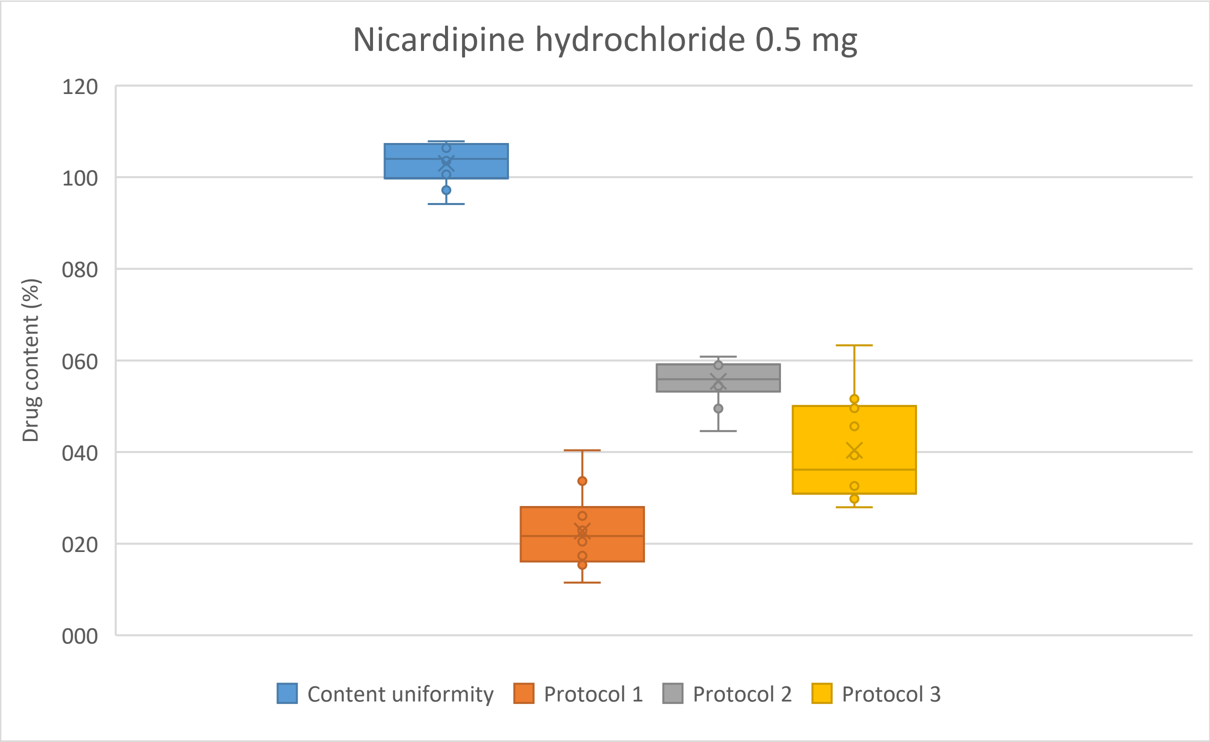

Figure S10. Content uniformity of Furosemide 2 mg and recovered drug content according to protocols 1, 2 and 3

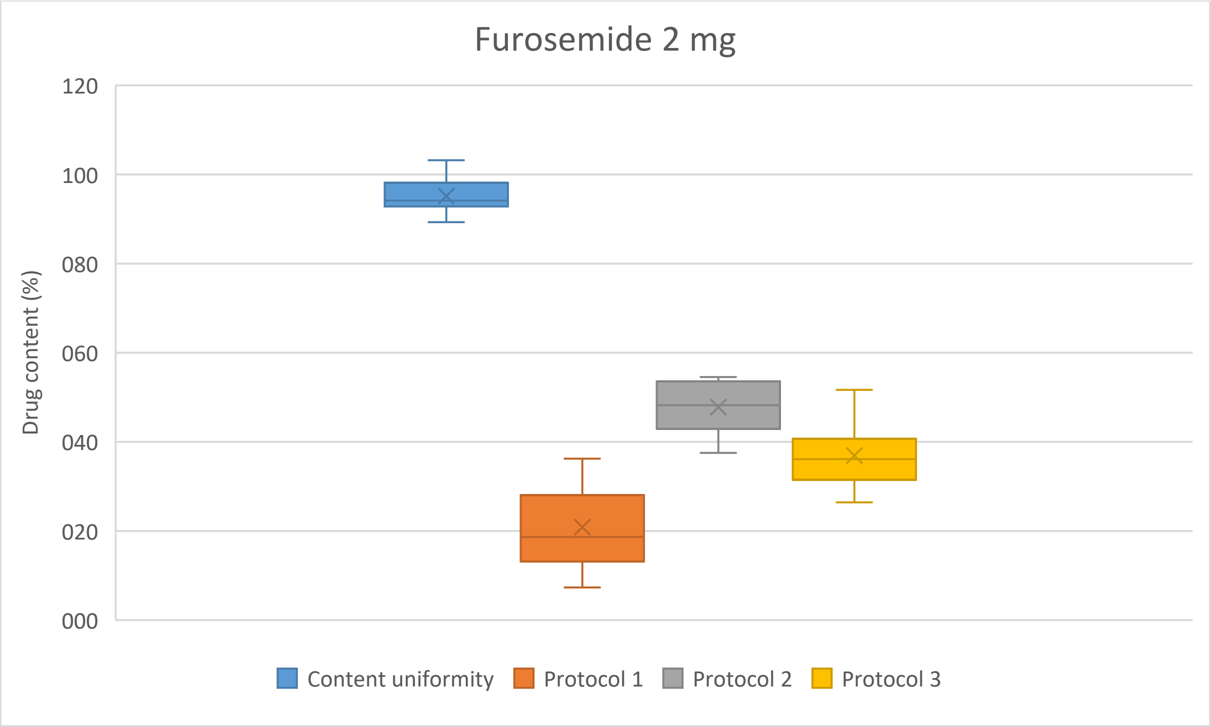

Table S1. Repeatability, intermediate precision and accuracy for furosemide, phenobarbital, sildenafil and ursodeoxycholic acid.

Samples and concentration	Repeatability (% RSD within-day) (n=15)	Intermediate precision (% RSD between-day) (n=18)	Accuracy (bias in %) (n=18)
Furosemide 0.18 $mg.mL^{-1}$	1.674	2.336	2.222
Furosemide 0.20 $mg.mL^{-1}$	1.554	2.684	4.250
Furosemide 0.22 $mg.mL^{-1}$	1.666	2.594	3.258
Phenobarbital 0.45 $mg.mL^{-1}$	0.737	1.481	2.346
Phenobarbital 0.50 $mg.mL^{-1}$	0.895	1.184	0.533
Phenobarbital 0.55 $mg.mL^{-1}$	1.198	1.272	1.212
Sildenafil citrate 35 $\mu g.mL^{-1}$	0.957	2.800	-0.704
Sildenafil citrate 70 $\mu g.mL^{-1}$	2.040	1.719	0.358
Sildenafil citrate 80 $\mu g.mL^{-1}$	1.012	1.664	2.522
Ursodeoxycholic acid 100 $\mu g.mL^{-1}$	0.609	1.725	-1.682
Ursodeoxycholic acid 300 $\mu g.mL^{-1}$	0.919	2.024	-3.333
Ursodeoxycholic acid 330 $\mu g.mL^{-1}$	1.473	1.426	0.839
